# Searching for bridges between psychopathology and real-world functioning in first-episode psychosis: a network analysis from the OPTiMiSE trial

**DOI:** 10.1192/j.eurpsy.2023.599

**Published:** 2023-07-19

**Authors:** F. Dal Santo, E. Fonseca-Pedrero, M. P. García-Portilla, L. González-Blanco, P. A. Sáiz, S. Galderisi, G. M. Giordano, J. Bobes

**Affiliations:** ^1^SESPA, University of Oviedo, CIBERSAM, ISPA, Oviedo; ^2^University of La Rioja, CIBERSAM, Logroño, Spain; ^3^University of Campania “Luigi Vanvitelli”, Naples, Italy

## Abstract

**Introduction:**

Network analysis has been used to explore the interplay between psychopathology and functioning in psychosis, but no study has used dedicated statistical techniques to focus on the bridge symptoms connecting these domains.

**Objectives:**

The current study aims to estimate the network of depressive, negative, and positive symptoms, general psychopathology, and real-world functioning in people with first-episode schizophrenia or schizophreniform disorder, focusing on bridge nodes.

**Methods:**

Baseline data from the OPTiMiSE trial were analysed. The sample included 446 participants (age 40.0±10.9 years, 70% males). The network was estimated with a Gaussian graphical model (GGM), using scores on individual items of the Positive and Negative Syndrome Scale (PANSS), the Calgary Depression Scale for Schizophrenia (CDSS), and the Personal and Social Performance (PSP) scale. Stability, strength centrality, expected influence (EI), predictability, and bridge centrality statistics were computed. The top 20% scoring nodes on bridge strength were selected as bridge nodes.

**Results:**

Nodes from different *rating scales* assessing similar psychopathological and functioning constructs tended to cluster together in the estimated network (Fig. 1). The most central nodes (EI) were Delusions, Emotional Withdrawal, Depression, and Depressed Mood. Bridge nodes included Depression, Conceptual Disorganisation, Active Social Avoidance, Delusions, Stereotyped Thinking, Poor Impulse Control, Guilty Feelings, Unusual Thought Content, and Hostility. Most of the bridge nodes belonged to the general psychopathology subscale of the PANSS. Depression (G6) was the bridge node with the highest value.

**Image:**

**Figure:**
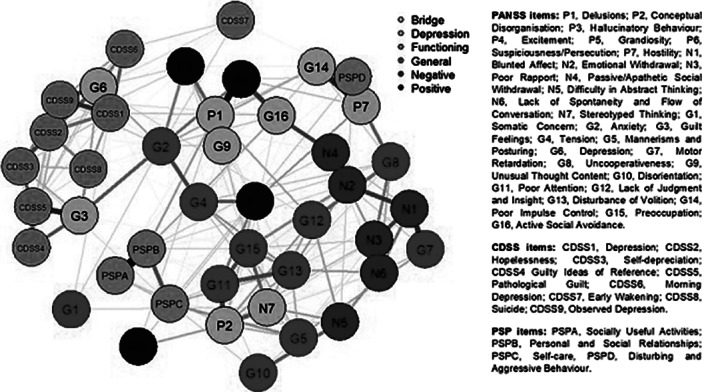

**Conclusions:**

The current study provides novel insights for understanding the complex phenotype of psychotic disorders and the mechanisms underlying the development and maintenance of comorbidity and functional impairment after psychosis onset.

**Disclosure of Interest:**

None Declared

